# An Aeromagnetic Compensation Method for Suppressing the Magnetic Interference Generated by Electric Current with Vector Magnetometer

**DOI:** 10.3390/s22166151

**Published:** 2022-08-17

**Authors:** Chao Zhang, Changping Du, Xiang Peng, Qi Han, Hong Guo

**Affiliations:** 1 State Key Laboratory of Advanced Optical Communication Systems and Networks, School of Electronics, Peking University, Beijing 100871, China; 2 Center for Quantum Information Technology, Peking University, Beijing 100871, China; 3 School of Computer Science and Technology, Harbin Institute of Technology, Harbin 150001, China

**Keywords:** aeromagnetic compensation, electric current magnetic interference, vector magnetometer, geomagnetic gradient interference, IGRF model

## Abstract

In aeromagnetic detection, the magnetic interference conducted by electric currents in onboard electronic (OBE) equipment is gradually being taken seriously with the development of aeromagnetic compensation technology. Here, we propose a compensation method based on the synthetically total magnetic field (STMF) measured by an onboard fluxgate vector magnetometer. In this method, a compensation model is firstly built to suppress the electric current magnetic interference (ECMI) which is jointly measured by a scalar magnetometer and a fluxgate vector magnetometer. The singular spectrum analysis (SSA) method is introduced to accurately extract the characteristic signal of the ECMI from the compensated STMF. In addition, in order to better suppress the geomagnetic gradient interference, the International Geomagnetic Reference Field (IGRF) model is introduced to modify the existing geomagnetic gradient compensation model. Based on these, a novel compensation model including the traditional aeromagnetic compensation model, modified geomagnetic gradient model, and ECMI compensation model is proposed. The results in the field experiment show that this model has better compensation performance than the TLG model, which is extended from the T–L compensation model.

## 1. Introduction

Aeromagnetic detection has been widely applied in geophysical exploration, geological mapping, leakage detection of oil and gas pipelines, shipwreck salvaging, and other fields in recent years [1,2,3,4,5]. Its working principle is to find the buried ferromagnetic object by analyzing the anomaly of the geomagnetic field, which is caused by the ferromagnetic object and is sensed by the airborne magnetometer. However, the airborne magnetometer also senses magnetic interference from the motion platform, geomagnetic gradient, and geomagnetic environmental background field when collecting the magnetic field of the object. The magnetic interference from the platform is larger than the object’s magnetic field because the magnetometer is very close to the fuselage. To suppress it, Tolls and Lawson proposed the T–L compensation model in 1950 [6]. In this model, the authors categorized the magnetic interference into three parts: permanent field, induced field, and eddy field. In Ref. [7], the interference is written as the form of 16-term compensation functions, each of which consists of a fixed compensation coefficient and a basis function, and a practical application method has also been provided. To solve the problem of multicollinearity in the equations constructed by the T–L model, Bickel proposed the small signal method by decomposing the direction cosine angle into the form of the main direction and its variation, obtaining the solution of the equations in 1979 [8]. With the improvement of computing power and the development of mathematical solution methods, more methods have been applied to accurately solve the equations, such as recursive least squares (RLS), truncated singular value decomposition, ridge estimation, and neural networks [9,10,11,12,13,14]. These methods can improve the compensation accuracy to a certain extent, but they still cannot satisfy the requirements of modern, high-precision aeromagnetic compensation.

The platform magnetic interference not only contains the traditional maneuver magnetic interference, but also contains electric current magnetic interference (ECMI) from in onboard electronic (OBE) equipment and magnetic interference caused by movable parts relative to the fuselage. The quality of aeromagnetic compensation can be improved through studying the sources of these additional magnetic interferences and forming the updated compensation models. In Ref. [15], the authors proposed to use electromagnetic compatibility technology to suppress the current magnetic interference of switch mode power supply on aircraft platform. In Ref. [16], the authors proposed compensation methods for the magnetic interference from the relative movable component and onboard electric current, respectively. In Ref. [17], the authors proposed a compensation method for the ECMI according to monitor the current change in every electronic equipment. However, the compensation effect of these models on the ECMI relies on the accuracy of the sensor used to monitor the electric current, and a large number of current sensors are needed to monitor all current drivers which can cause magnetic interference. Thus, the compensation accuracy of these models has been limited by the precisions of current sensors and adaptive compensation methods. In addition, magnetic sensors are also affected by temperature. In Ref. [18], the authors prepared hybrid reduced graphene oxide nanosheets that have large magnetoresistance at a low magnetic field at room temperature and used in wireless magnetic field sensors for quick detection of low electromagnetic radiation. In Ref. [19], the authors proposed a novel method for the temperature-compensated inductance–to–frequency converter, which can be used in magnetic sensors.

In this paper, a new method to compensate the ECMI based on a fluxgate vector magnetometer is proposed. In practice, there are two different magnetometers in the airborne detection system. One is a scalar magnetometer which is far from the aircraft and used to measure the scalar target magnetic signal, the other is a vector magnetometer which is near the aircraft body and used to obtain the direction cosine of the geomagnetic field in the reference system of the airborne platform. Thus, both magnetometers will collect scalar and vector ECMI at the same time. The ECMI measured by the scalar magnetometer can be expressed by projecting the vector field of the ECMI to the direction of the geomagnetic field [16]. Based on this, this paper infers the compensation model of the ECMI, and the model is an expression including the compensated synthetically total magnetic field (STMF) of the ECMI, direction cosine of geomagnetic field and constant coefficient to be estimated. In order to obtain the accurate ECMI characteristic signal from the STMF measured by the onboard fluxgate vector magnetometer, we apply a decomposition method to extract the ECMI characteristic signal from the residual magnetic field, which is the STMF after suppressing the platform maneuver magnetic interference and geomagnetic gradient interference. Common decomposition techniques include wavelet transform (WT), ensemble empirical mode decomposition (EEMD), singular spectrum analysis (SSA) and others [20,21,22]. An appropriate wavelet basis function needs to be firstly determined in the WT, but it is difficult to find a proper wavelet basis function to accurately extract the characteristic signal of the ECMI [23]. EEMD is an improved version of empirical mode decomposition (EMD) that can decompose nonlinear and non-stationary signals. However, EEMD has the disadvantage of edge effect and certain noise in each component after decomposition [24]. SSA is a powerful tool and currently developed for dealing with nonlinear and non-stationary time series without the edge effect or requiring prior information to find an appropriate basis function. It can also be combined with elements of classical time-series analysis, multivariate statistics, and signal processing to process complicated signals, and is used in many fields [25]. Therefore, SSA is introduced to extract the characteristic signal of ECMI in this paper. In addition, the International Geomagnetic Reference Field (IGRF) model is also brought in to form a novel compensation model for geomagnetic gradient interference by combining with the first-order geomagnetic gradient compensation model. Thus far, a novel compensation model can be constructed by combining the traditional magnetic interference compensation model, the ECMI compensation model, and the modified geomagnetic gradient compensation model.

In this paper, we first deduce the relationship between the ECMI measured by the scalar magnetometer at the far end of the fuselage and the synthetic ECMI field measured by the vector magnetometer near the fuselage. Then, we introduce the SSA method to accurately extract the characteristic signal of ECMI from the compensated STMF. Next, a new geomagnetic gradient interference compensation model is constructed by combining the IGRF model and the previous first–order linear model. Finally, the aeromagnetic compensation model proposed in this paper is summarized. Some field experiments are arranged to illustrate the effectiveness of this compensation method.

## 2. Analysis and Methods

In the aeromagnetic detection, the total magnetic field $B_{t}$ measured by the magnetometer includes the target signal $B_{M}$, the platform maneuver magnetic interference $B_{TL}$, the geomagnetic gradient interference field $B_{E}$, and the ECMI $B_{C}$. Thus, $B_{t}$ can be expressed as

(1) $$B_{t}=B_{M}+B_{TL}+B_{E}+B_{C}.$$

In order to better detect the target signal $B_{M}$, the three kinds of magnetic interference need to be well-suppressed.

### 2.1. Compensation Model for Platform Maneuver Magnetic Interference

Tolles and Lawson regarded the platform as a conjoined and rigid whole, and believed that the maneuver magnetic interference comes from the permanent magnetic field, induced magnetic field, and eddy magnetic field of the platform. Therefore, a compensation model was constructed according to the physical analysis of the generation of three magnetic fields to suppress the platform maneuver magnetic interference. This compensation model is usually called the T–L compensation model, which will be briefly introduced below.

**Figure 1:**
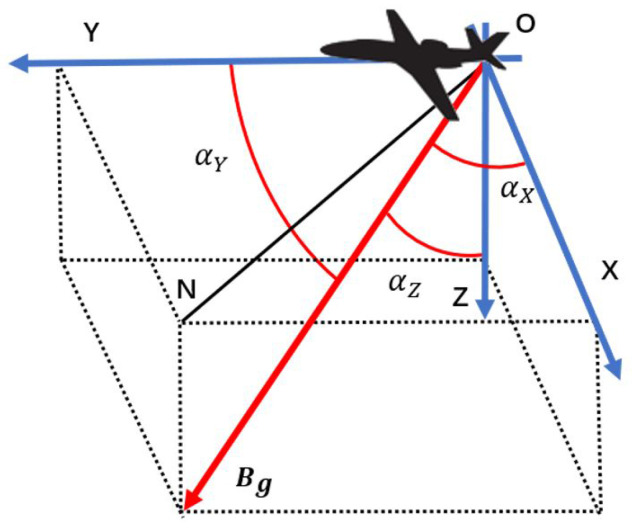
The reference coordinate system defined in this paper.

In this paper, a coordinate system is established with the position of the onboard magnetometer as the origin O, as shown in Figure 1. The Y-axis points to the nose along the direction of the fuselage, the Z-axis is perpendicular to the fuselage downward, and the X-axis, Y-axis, and Z-axis follow the right-hand rule. Among them, N is the geomagnetic north, $B_{g}$ is the vector geomagnetic field, and $\alpha_{X}$, $\alpha_{Y}$ and $\alpha_{Z}$ are the angles between the geomagnetic field $B_{g}$ and the three axes of the platform coordinate system.

According to the T–L model, the platform maneuver magnetic interference model consists of three permanent field components, five induced field components, and eight eddy field components, each of which is expressed as a fixed compensation coefficient and a corresponding basis function [26]

(2) $$B_{TL}=\sum_{i=1}^{16}a_{i}\mu_{i},$$

where $a_{i}$(i=1…16) is the compensation coefficient to be estimated, and $\mu_{i}$(i=1…16) is the relevant basis function, $\mu_{1}=\cos\alpha_{X}$, $\mu_{2}=\cos\alpha_{Y}$, $\mu_{3}=\cos\alpha_{Z}$, $\mu_{4}=B_{g}\cos\alpha_{X}\cos\alpha_{X}$, $\mu_{5}=B_{g}\cos\alpha_{X}\cos\alpha_{Y}$, $\mu_{6}=B_{g}\cos\alpha_{X}\cos\alpha_{Z}$, $\mu_{7}=B_{g}\cos\alpha_{Y}\cos\alpha_{Y}$, $\mu_{8}=B_{g}\cos\alpha_{Y}\cos\alpha_{Z}$, $\mu_{9}=B_{g}\cos\alpha_{X}{\left(\cos\alpha_{X}\right)}^{{}^{\prime }}$, $\mu_{10}=B_{g}\cos\alpha_{X}{\left(\cos\alpha_{Y}\right)}^{{}^{\prime }}$, $\mu_{11}=B_{g}\cos\alpha_{X}{\left(\cos\alpha_{Z}\right)}^{{}^{\prime }}$, $\mu_{12}=B_{g}\cos\alpha_{Y}{\left(\cos\alpha_{X}\right)}^{{}^{\prime }}$, $\mu_{13}=B_{g}\cos\alpha_{Y}{\left(\cos\alpha_{Y}\right)}^{{}^{\prime }}$, $\mu_{14}=B_{g}\cos\alpha_{Y}{\left(\cos\alpha_{Z}\right)}^{{}^{\prime }}$, $\mu_{15}=B_{g}\cos\alpha_{Z}{\left(\cos\alpha_{X}\right)}^{{}^{\prime }}$, $\mu_{16}=B_{g}\cos\alpha_{Z}{\left(\cos\alpha_{Y}\right)}^{{}^{\prime }}$, where $B_{g}=\left|B_{g}\right|$, $\cos\alpha_{X}$, $\cos\alpha_{Y}$ and $\cos\alpha_{Z}$ denote the cosine value of $\alpha_{X}$, $\alpha_{Y}$ and $\alpha_{Z}$, respectively. In addition, they also represent the direction cosine of $B_{g}$ in the coordinate system. ${\left(\cos\alpha_{X}\right)}^{{}^{\prime }}$, ${\left(\cos\alpha_{Y}\right)}^{{}^{\prime }}$ and ${\left(\cos\alpha_{Z}\right)}^{{}^{\prime }}$ are the time derivatives of $\cos\alpha_{X}$, $\cos\alpha_{Y}$ and $\cos\alpha_{Z}$, respectively, which are defined as

(3) $${\left(\cos M\right)}^{{}^{\prime }}=\frac{d\cos M(t)}{dt},$$

where *M* represents $\alpha_{X}$, $\alpha_{Y}$, or $\alpha_{Z}$, and varies with time.

The traditional aeromagnetic compensation methods do not consider the effect of the geomagnetic gradient interference and assume that the geomagnetic field is a constant. However, in practice, a large geomagnetic gradient interference will be introduced due to the non-stationary motion of the platform, which seriously affects the solution of the compensation coefficient and decreases the quality of aeromagnetic detection data. In Ref. [27], to suppress the geomagnetic gradient interference, the authors used the position information (i.e., longitude, latitude, and altitude) of the airborne platform to build a first–order Taylor polynomial compensation model and added it to the T–L compensation model to form the TLG model, which can be expressed as

(4) $$B_{TLG}=B_{TL}+B_{G}=\sum_{i=1}^{16}a_{i}\mu_{i}+\sum_{j=1}^{3}b_{j}g_{j},$$

where $b_{j}$(j=1,2,3) is the coefficient to be estimated, and $g_{j}$(j=1,2,3) is the location information of the platform, representing longitude, latitude, and altitude, respectively.

### 2.2. Compensation Model for the Magnetic Interference Generated by the Electric Current

#### 2.2.1. Analysis

The electric current in electronic systems on the motion platform will generate a magnetic interference field, and the magnetic field is related to the magnitude and direction of the electric current. In aeromagnetic detection, the detection target is usually in a very low frequency band (<1 Hz), so this paper only cares about the magnetic field in the low frequency band. The frequency range of ECMI we concern is lower than 0.1 Hz, and it can be thought be generated by quasi–static current. Thus, the magnetic field generated by quasi-static electric current in on-board electronic systems can be estimated by using the Biot–Savart law, which is usually used to calculate the static magnetic field.

Considering that the aircraft platform requires high security, the cables inside it need to be strictly routed and “node–type” fixed. The position of the cable is therefore fixed throughout the entire flight. The magnetic interference generated by the cable will attenuate in a cubic form with the distance from the observation, so the magnetic interference field caused by some cables that are far away from the magnetometer is negligible, and the cable that is closer to the magnetometer is called as the effective interference segment. Let *a* and *b* be the start and end points of the effective interference segment, respectively. Therefore, we make equivalent the effective interference segment from *a* to *b* as a finite length current–carrying wire. In addition, the magnetic interference generated by it can be regarded as the superposition of the magnetic interference generated by many different pieces of current element Idl in *a* to *b*. According to the Biot–-Savart law [28], the vector ECMI in the place where the scalar magnetometer is installed can be expressed as

(5) $$B_{Id}\left(r_{s},t\right)=-\frac{\mu_{0}}{4\pi }\int_{a}^{b}\frac{R_{s}}{R_{s}^{3}}\times I\left(t\right)dl,$$

where $R_{s}=r_{s}-r$, $R_{s}=\left|R_{s}\right|$, in which $r_{s}$ is the vector position of scalar magnetometer, r is the vector position of the current element and $\mu_{0}$ is the vacuum permeability. Let *C*$=-\mu_{0}/\left(4\pi \right)$ and $K_{s}=\int_{a}^{b}R_{s}/R_{s}^{3}\times dl=\left(k_{s1},k_{s2},k_{s3}\right)$, so $B_{Id}$ can be shortly written as

(6) $$B_{Id}=CIK_{s}.$$

From Equation (2), $B_{g}/\left|B_{g}\right|=\left(\mu_{1},\mu_{2},\mu_{3}\right)$. Thus, the scalar magnetic field $B_{Id}$ is the projection of the vector expression onto the direction of the geomagnetic field, which can be expressed as

(7) $$B_{Id}=B_{Id}\cdot \frac{B_{g}}{\left|B_{g}\right|}=CI\left(k_{s1}\mu_{1}+k_{s2}\mu_{2}+k_{s3}\mu_{3}\right).$$

In the aeromagnetic detection, the vector magnetometer is installed in the place closer to the fuselage and can also obtain the ECMI signal at the same time. Thus, the vector ECMI measured by the onboard fluxgate vector magnetometer can be written as

(8) $$B_{Cv}=CIK_{v},$$

where $K_{v}=\int_{a}^{b}R_{v}/R_{v}^{3}\times dl=\left(k_{v1},k_{v2},k_{v3}\right)$, in which $R_{v}=r_{v}-r$ and $r_{v}$ is the vector position of fluxgate magnetometer. Thus, the STMF measured by the onboard fluxgate vector magnetometer can be expressed as

(9) $$B_{Cv}=CI\sqrt{k_{v1}^{2}+k_{v2}^{2}+k_{v3}^{2}}.$$

Take the above formula into Equation (7), and the scalar magnetic field caused by the electric current can be rewritten as

(10) $$\begin{array}{cc} B_{Id} & =\frac{B_{Cv}}{\sqrt{k_{v1}^{2}+k_{v2}^{2}+k_{v3}^{2}}}\left(k_{s1}\mu_{1}+k_{s2}\mu_{2}+k_{s3}\mu_{3}\right). \end{array}$$

Because the positions of the scalar magnetometer and the fluxgate vector magnetometer are fixed, the distance between the magnetometers and the current element remains constant throughout the whole flight. Thus, Equation (10) can be written as

(11) $$B_{Id}=B_{Cv}\left(p_{1}\mu_{1}+p_{2}\mu_{2}+p_{3}\mu_{3}\right),$$

where $p_{1}$, $p_{2}$, and $p_{3}$ are the three constant coefficients. As can be seen from the above formula, $B_{Id}$ can be linearly represented by $B_{Cv}$. However, the vector fluxgate magnetometer is also affected by platform maneuver magnetic interference and geomagnetic gradient interference while obtaining $B_{Cv}$. Thus, the processing methods need to be introduced to suppress those magnetic interference to obtain relatively accurate ECMI. Then, we use an extraction method to obtain the characteristic signal $h_{c}$ from the compensated magnetic field, which is directly proportional to the ECMI measured by the fluxgate vector magnetometer. Finally, taking $h_{c}$ into the Equation (11), and the $B_{C}$ of predicted ECMI can be expressed as

(12) $$B_{C}=h_{c}\left(q_{1}\mu_{1}+q_{2}\mu_{2}+q_{3}\mu_{3}\right),$$

where $q_{1}$, $q_{2}$, and $q_{3}$ are the coefficients to be estimated.

#### 2.2.2. Extraction Method of the Characteristic Signal of ECMI

The extraction method is very important when compensating the ECMI, and common extraction methods contain WT, EEMD, and SSA. Here, the three methods will be compared and the best will be chosen as the effective extraction method.

(1) Wavelet Transform

WT can locally analyze the data in time (space) frequency, gradually refine the signal through scale transformation and translation operation, and finally realize the time subdivision of high frequency and frequency subdivision of low frequency.

Therefore, WT can effectively decompose the non-stationary signal into signals of different frequency bands, but because the wavelet basis function is not unique, the results obtained by decomposing the same signal with different wavelet basis functions will be different. Therefore, WT needs prior knowledge to determine the optimal wavelet basis. This will be adverse to the establishment of ECMI compensation model.

(2) Ensemble Empirical Mode Decomposition

EEMD is an improved version of EMD and can well decompose the nonlinear and non–stationary signal to give a clear analysis. EMD can adaptively decompose the non–stationary original signal into intrinsic mode function (IMF) of different frequency bands, and it avoids the selection of basis functions. However, this technique suffers from mode mixing problem, i.e., a component of a similar scale will reside in different IMFs. To alleviate from this problem, the EEMD technique has been proposed in [29]. It takes advantage of the uniform distribution of the power spectral density of Gaussian white noise, and adds the Gaussian white noise into the signal to be decomposed.

EEMD ameliorates the problem of mode aliasing existing in EMD to some extent, but does not improve the problem of the edge effect and brings in some noise into each component after decomposition, so that the decomposition result will produce a certain degree of distortion [24]. These factors are not conducive to the extraction of ECMI.

(3) Singular Spectrum Analysis

SSA is a new decomposing method used to deal with nonlinear and non-stationary time series. The SSA algorithm first builds a trajectory matrix through a sliding window, and then uses singular value decomposition to decompose the trajectory matrix into the form of singular values and eigenvectors, and then classifies the singular values according to the amplitude of the value. Finally, it uses the classified singular values and corresponding vectors to reconstruct the data and then effectively decomposes the original single-channel sequence data into independent components such as slow variation trend, oscillation, and noise [25]. SSA can not only avoid the selection of basis functions, but also avoid the pollution of white noise to the signal and edge effect. Compared with the other two decomposition methods, this method is more conducive to extracting the ECMI. Therefore, we will use SSA to extract $h_{c}$ from the compensated STMF $B_{re_{-}flux}$ measured by the fluxgate vector magnetometer, and its process is described below.

Step 1. Let *N* be the length of sequence $B_{re_{-}flux}$, select the appropriate window length *L*(L<N/2), and set K=N−L+1. Arrange $B_{re_{-}flux}$ to obtain the trajectory matrix X, which is expressed as

(13) $$X=\left[\begin{array}{cccc} x_{1} & x_{2} & \cdots & x_{K}\\ x_{2} & x_{3} & \cdots & x_{K+1}\\ \vdots & \vdots & \; & \vdots \\ x_{L} & x_{L+1} & \cdots & x_{N} \end{array}\right].$$

Step 2. The covariance matrix of X is $S=XX^{T}$. Obtain the eigenvalues $\lambda_{1}$, $\lambda_{2}\ldots \lambda_{L}$ of S and the corresponding eigenvectors $\delta_{1}$, $\delta_{2}\ldots \delta_{L}$ by eigenvalue decomposition. Here, the eigenvalues are arranged in descending order. Let $v_{m}=X^{T}\delta_{m}/\sqrt{\lambda_{m}}$(m=1,2⋯L). Then, the *m*th component of the trajectory matrix X can be obtained, which is expressed as

(14) $$X_{m}=\sqrt{\lambda_{m}}\delta_{m}v_{m}^{T}=\delta_{m}\delta_{m}^{T}X.$$

Through the above steps, *L* components can be obtained.

Step 3. From the above, $X_{m}$ corresponds to the eigenvalue $\lambda_{m}$. Divide the above *L* components into *c* groups (c=2 in this paper) using a certain method. Then, add the components in the same group to obtain a new L×K matrix $S_{j}$(j=1,2). Here, we group the *L* components by calculating the contribution rate *P* of the eigenvalues. *P* is defined as

(15) $$P=\frac{\sum_{i=1}^{d}\lambda_{i}}{\sum_{j=1}^{L}\lambda_{j}}\times 100\%,$$

where d≤L. When P≥85%, the first *d* components in the *L* components will be divided into the first group, and the remaining components will be divided into the second group.

Step 4. The new L×K matrix $S_{j}$ can be transformed into a one-dimensional sequence $\left[s_{1}^{*},s_{2}^{*},\cdots ,s_{N}^{*}\right]$ by taking the diagonal averaging method, as described below. $s_{ij}$ is the element in the *i*th row and the *j*th column in $S_{j}$,

(16) $$s_{n}^{*}=\left\{\begin{array}{cc} \frac{1}{n}\sum_{m=1}^{n}s_{m,n-m+1} & \left(1\leq n<L\right)\\ \frac{1}{L}\sum_{m=1}^{L}s_{m,n-m+1} & \left(L\leq n<K\right)\\ \frac{1}{N-n+1}\sum_{m=n-K+1}^{N-K+1}s_{m,n-m+1} & \left(K\leq n<N\right) \end{array}\right.,$$

where n=1,2⋯N. Finally, $h_{c}$ is obtained by distinguishing the magnitude of the decomposed signals.

### 2.3. Modified Compensation Model for Geomagnetic Gradient Interference

The geomagnetic gradient interference compensation model in the TLG model cannot fully describe the complex geomagnetic gradient interference due to its low-order linearity [30]. The compensation accuracy of this model is low in a large working area or an area where the geomagnetic gradient is not uniform. To solve this problem, we combine the IGRF model with the linear model to construct a novel compensation model for the geomagnetic gradient interference, which will be called the TLGI model. The expression is written as

(17) $$B_{TLGI}=B_{TLG}+B_{IGRF}=\sum_{i=1}^{16}a_{i}\mu_{i}+\sum_{i=1}^{3}b_{i}g_{i}+cf,$$

where *f* is the predicted geomagnetic field obtained by using the IGRF model with the position information (i.e., longitude, latitude, and altitude) of the airborne platform. *c* is the coefficient to be estimated.

### 2.4. A Novel Compensation Model

Combining Equations (12) and (17), a new compensation model TLGIC including the platform maneuver magnetic interference compensation model, geomagnetic gradient interference compensation model, and the ECMI compensation model can be expressed as

(18) $$B_{TLGIC}=B_{TL}+B_{G}+B_{IGRF}+B_{C}=\sum_{i=1}^{16}a_{i}\mu_{i}+\sum_{i=1}^{3}b_{i}g_{i}+cf+\sum_{i=1}^{3}q_{i}h_{c}\mu_{i}.$$

The above formula can be rearranged as

(19) $$B_{TLGIC}=J\cdot C,$$

where J is a row vector consisting of $\mu_{i}$, $g_{i}$, *f* and $h_{c}u_{i}$, and C is a column vector consisting of the compensation coefficients $a_{i}$, $b_{i}$, *c*, and $q_{i}$. The compensation coefficients will be solved in a calibration flight where three groups of maneuver actions are executed. In the flight, the aircraft usually maneuvers at a certain frequency. A bandpass filter with a narrow bandwidth in which most of the maneuver magnetic interference and limited other magnetic interference are contained will be applied to the two sides of the equations formed by the TLGIC compensation model to equalize the equations when solving the coefficients.

In summary, the key steps of using the TLGIC model for aeromagnetic compensation are as follows:

Step 1. The total magnetic field $B_{flux}$ is synthesized by using the data collected by the fluxgate vector magnetometer, and the basic functions of TLG compensation model are constructed with the magnetic data measured by the fluxgate vector magnetometer, and the position information measured by the Beidou satellite navigation system. A Butterworth bandpass filter with a frequency band of 0.06∼0.6 Hz is applied to the both ends of the compensation equations, and then the RLS method is used to obtain the compensation coefficients. Finally, the residual magnetic field can be obtained by subtracting the magnetic field calculated by the compensation coefficients and basis functions from $B_{flux}$.

Step 2. The SSA algorithm is applied to extract the characteristic signal of ECMI $h_{c}$ from the residual magnetic field in the step 1 and construct the compensation model of ECMI $B_{C}$.

Step 3. The TLGIC model is constructed using the TLG compensation model, the IGRF model and the compensation model $B_{C}$, and then uses the same method as that in step 1 to solve the compensation coefficients C.

Step 4. In the detection flight, the TLGIC compensation model and the compensation coefficients obtained in the above steps will be used to compensate the measurement magnetic data in real time.

## 3. Field Experiment

In order to verify the effectiveness of the method proposed in this paper, some field experiments with a fixed-wing unmanned aerial vehicle were carried out in the field, where the geomagnetic background field is magnetically quiet. In the experiment, to obtain the accurate compensation coefficients, let the platform perform roll, pitch, and yaw maneuvers in the directions of east, south, west, and north in a fixed period. The flying altitude is controlled to avoid magnetic interference caused by local geology. In addition, the optically pumped magnetometer with ${}^{4}\mathrm{He}$ atoms with accuracy <2.5 nT and the range of 20∼100 μT is used for measuring the scalar magnetic field, and its principle can be seen in [31]. A fluxgate vector magnetometer with accuracy <10 nT and a range of −100∼100 μT is used to obtain the motion attitude of the platform. In addition, the Beidou satellite navigation system is used to obtain the position information of the platform in real time. Two laps of calibration flights are arranged in the experiment, in which the first lap is used to obtain the compensation coefficients, and the second lap is to verify the applicability of the compensation coefficients.

**Figure 2:**
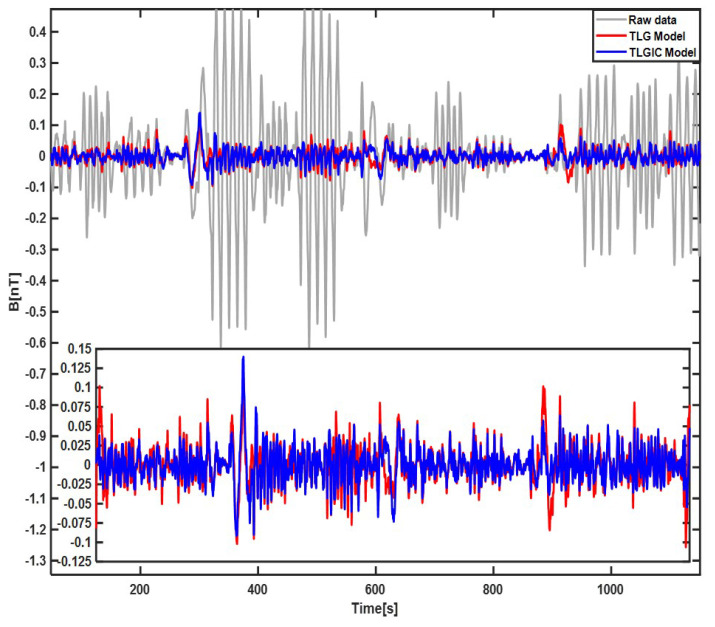
Compensation results of the first–lap flight data using the TLG model and TLGIC model.

**Figure 3:**
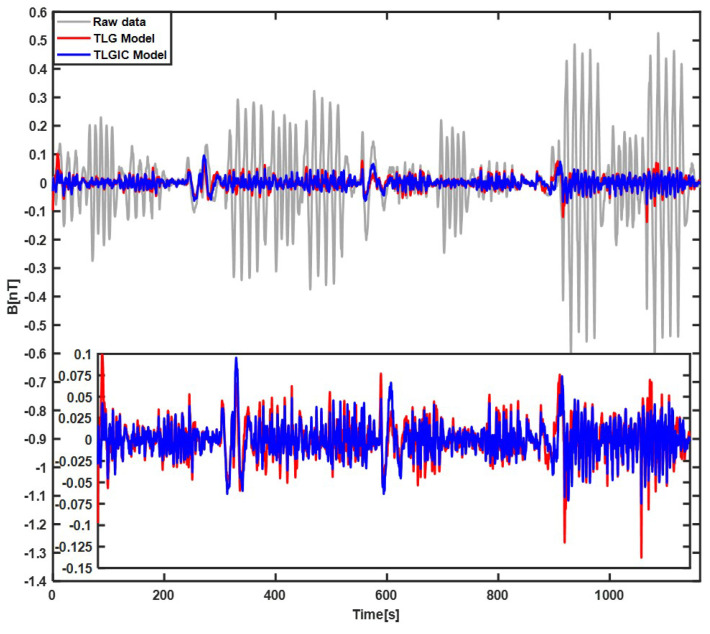
Compensation results of the second–lap flight data by the compensation coefficients calculated in the first–lap flight.

The compensation results of the first lap flight data using the two compensation models TLG and TLGIC are shown in Figure 2. The grey line is the filtered original magnetic field, the red line is the filtered compensation result of the TLG model, and the blue line is the filtered compensation result of the TLGIC model. The results show that two models can both obviously compensate the maneuver magnetic interference, but the compensation effect of TLGIC model is better. Then, the coefficients of two models solved in the first lap are used for the magnetic data of the second lap to verify their universality, and the results are shown in Figure 3. We can clearly see that the compensation effect of the TLGIC model is still better than that of the TLG model.

**Figure 4:**
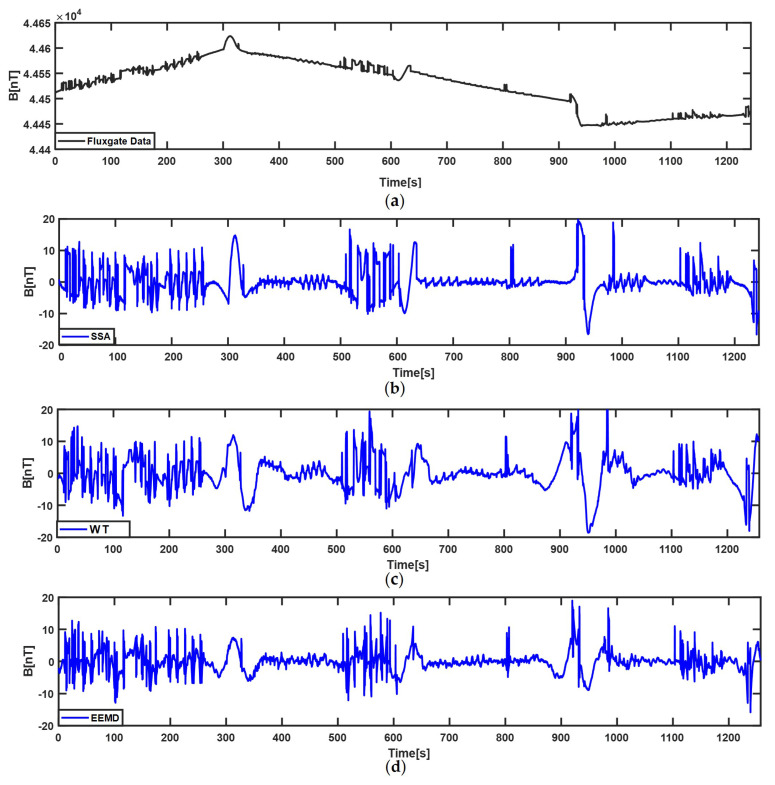
The extraction results of three methods for ECMI of the first–lap. (**a**) the compensated STMF of the fluxgate vector magnetometer; (**b**) the characteristic signal of ECMI extracted by SSA; (**c**) the characteristic signal of ECMI extracted by WT; (**d**) the characteristic signal of ECMI extracted by EEMD.

**Figure 5:**
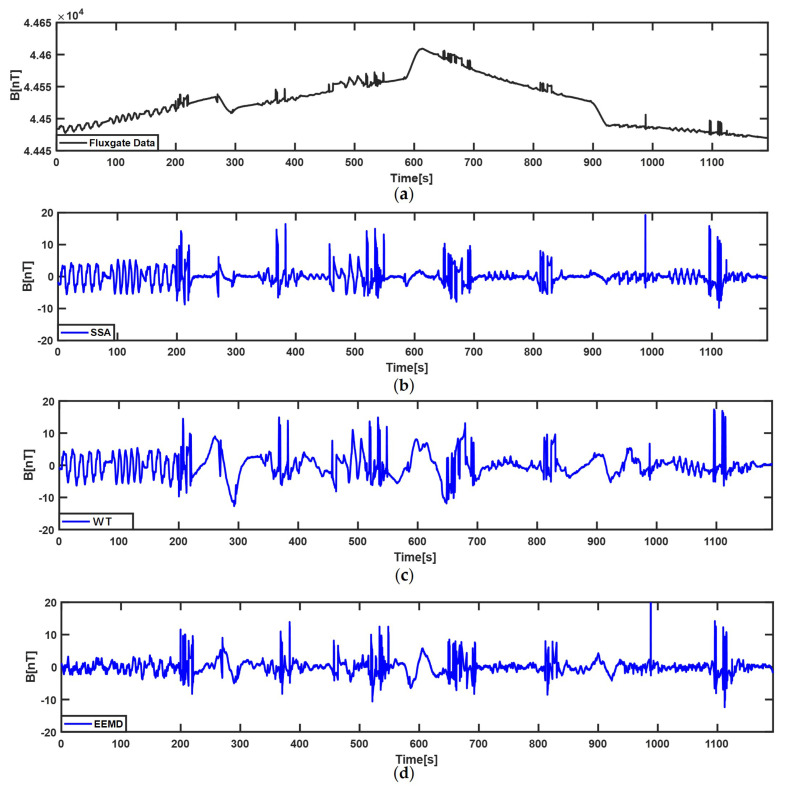
The extraction results of three methods for ECMI of the second–lap. (**a**) the compensated STMF of the fluxgate vector magnetometer; (**b**) the characteristic signal of ECMI extracted by SSA; (**c**) the characteristic signal of ECMI extracted by WT; (**d**) the characteristic signal of ECMI extracted by EEMD.

Figure 4 and Figure 5 show the decomposition results of the compensated STMF that come from the fluxgate vector magnetometer in two calibration flights using WT, EEMD, and SSA algorithms. In the two figures, (a) is the compensated STMF; (b), (c), and (d) are the extraction results of SSA, WT, and EEMD algorithms, respectively. In the SSA algorithm, the window length is set to 100 considering the maximum width of the ECMI signal. As can be seen from Figure 4b and Figure 5b, the SSA algorithm can well extract the characteristic signal of the ECMI and retain more details of the ECMI. In WT, Daubechies wavelet series is a series of compact support and orthonormal wavelet functions [32], of which the Daubechies-4 (db4) wavelet is often used for characteristic signal extraction in non-stationary time series [33,34,35], so it is selected as the decomposition basis function in this paper. As well as the number of decomposition layers being set to 10, the higher-frequency noise components and low-frequency trend components are removed, and the signals of the third to eighth decomposition layers are superimposed to obtain the ECMI characteristic signal. In EEMD, the signal is also decomposed into ten IMFs, and the fourth to eighth IMF components are selected to reconstruct a new signal. By comparing the three subfigures (b), (c), and (d) in Figure 4 and Figure 5, it is obvious that SSA can more effectively decompose out the characteristic signal of the ECMI from the compensated STMF, and the extraction result contains the complete characteristic signal of the ECMI, especially in the turn. Although WT and EEMD can also extract the ECMI characteristic signal, their extraction results distort the characteristic of the ECMI to certain extent, which also explains why the compensation result of TLGIC_SSA is the best one.

Here, we define the three compensation models constructed by combining three decomposing methods with the TLGIC compensation model as TLGIC_SSA, TLGIC_WT, and TLGIC_EEMD. To better show the compensation performances of the three methods, three indicators, Figure of Merit (FOM), Standard Deviation (STD) and Improvement Ratio (IR) [14,36], are used to evaluate their compensation results. The evaluation results are shown in Table 1 and Table 2, fully showing that the performance of several compensation algorithms related to the TLGIC model is obviously better than that of the TLG model, and the compensation performance of the TLGIC_SSA model is the best one, which is consistent with our expected results. In the first compensation case, by comparing the compensated results of the TLGIC_SSA model with that of the TLG model, STD reduces by 36.36%, IR increases by 57.12%, and FOM reduces by 24.54%. In the second compensation case, STD reduces by 35.48%, IR increases by 54.99%, and FOM reduces by 27.10%.

**Figure 6:**
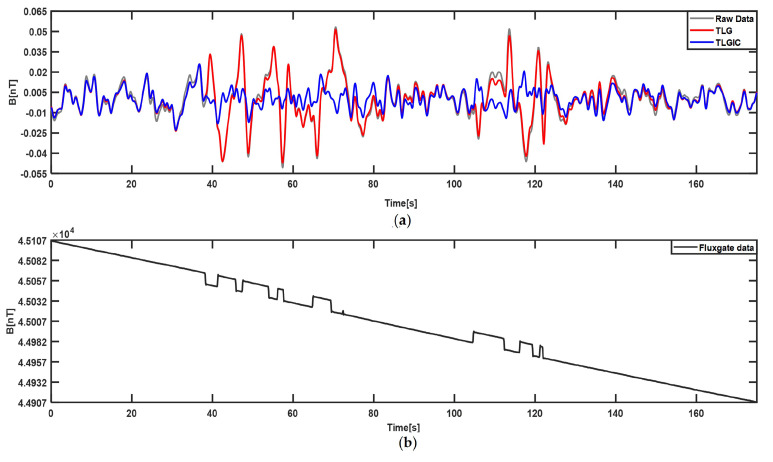
Compensation results of measured magnetic data in low–altitude flight by utilizing TLG and TLGIC_SSA compensation models. (**a**) low–altitude flight data before and after compensation; (**b**) compensated total magnetic field data measured by the fluxgate vector magnetometer.

Furthermore, in order to verify the compensation effect of the proposed algorithm in actual application, this paper also uses the compensation coefficients obtained by the two models in the first-lap to compensate the data of low-altitude flight, and the results are shown in Figure 6a. The compensated total magnetic field measured by the fluxgate vector magnetometer is plotted in Figure 6b to show the appearance time of the ECMI during flight and to make an amplitude comparison between the ECMI measured by the scalar magnetometer and the fluxgate vector magnetometer. Figure 6 shows that the ECMI in the magnetic data measured by the fluxgate vector magnetometer presents “gate”-shaped signal and its Peak-to-Peak Value (PPV) is about 25 nT, while the PPV in the scalar magnetometer is about 0.1 nT, and it is far smaller than that in the fluxgate vector magnetometer. In addition, Table 3 shows that the PPV of the compensation result of the TLGIC_SSA model decreased by 68% compared with that of TLG compensation model, and this illustrates that the TLGIC_SSA model can better suppress the ECMI of the platform.

## 4. Conclusions

In this paper, we propose a new aeromagnetic compensation model named the TLGIC model, including an ECMI compensation model, a modified geomagnetic gradient compensation model, and a T–L compensation model. In the ECMI compensation model, the relationship is first inferred between the ECMI measured by the scalar magnetometer mounted away from the fuselage and measured by a fluxgate vector magnetometer placed near the platform body, and a compensation model is constructed according to the relationship. In addition, the SSA algorithm is introduced to accurately extract the characteristic signal of ECMI from the compensated total magnetic field measured by the vector magnetometer. At the same time, considering the limitations in real applications of the first-order linear geomagnetic gradient compensation model, the IGRF model is introduced to combine with the first-order linear geomagnetic gradient compensation model to form a modified compensation model. Some field experiments have been arranged to verify that the new compensation model proposed in this paper has higher compensation accuracy than the traditional compensation model.

## Figures and Tables

**Table 1:** Compensation results for the first–lap flight data.

Model	STD (nT)	IR	FOM (nT)	IR
Raw	0.151	∖	6.387	∖
TLG	0.033	4.576	1.483	4.307
TLGIC_WT	0.022	6.864	1.175	5.436
TLGIC_EEMD	0.023	6.565	1.202	5.314
TLGIC_SSA	0.021	7.190	1.119	5.708

**Table 2:** Compensation results of the second–lap flight data by utilizing the compensation coefficients calculated by the first–lap data.

Model	STD (nT)	IR	FOM (nT)	IR
Raw	0.146	∖	6.376	∖
TLG	0.031	4.710	1.332	4.787
TLGIC_WT	0.023	6.348	1.166	5.468
TLGIC_EEMD	0.024	6.083	1.220	5.226
TLGIC_SSA	0.020	7.300	0.971	6.566

**Table 3:** Compensation results during a low-altitude flight.

Model	STD (nT)	PPV (nT)
Raw	0.015	0.104
TLG	0.014	0.098
TLGIC_SSA	0.007	0.036

## Data Availability

The data that support the findings of this study are available from the corresponding author, upon reasonable request.
